# Metachronous Renal Cell Carcinoma: A Rare Presentation of Adrenal Crisis

**DOI:** 10.7759/cureus.15965

**Published:** 2021-06-27

**Authors:** Andrew V Doodnauth, Syed Hamza Bin Waqar, Miriam M Klar, Zohra R Malik, Samy I McFarlane

**Affiliations:** 1 Internal Medicine, State University of New York Downstate Health Sciences University, Brooklyn, USA; 2 Internal Medicine, St. John's Episcopal Hospital, Far Rockaway, USA

**Keywords:** metastasis, renal cell carcinoma (rcc), adrenal glands, adrenal insufficiency, radical nephrectomy

## Abstract

Responsible for 2% of global cancer diagnoses, renal cell carcinoma (RCC) can metastasize to almost every organ system; however, metastasis to the contralateral adrenal gland is extremely rare. We report the case of a 59-year-old male who presented with atypical chest pain and altered mental status. The patient developed hypotension, with hyponatremia raising concern for adrenal insufficiency (AI). We confirmed a diagnosis of AI secondary to adrenal metastasis in the setting of radical nephrectomy with ipsilateral adrenalectomy, and the patient’s symptoms resolved with adequate treatment. This report emphasizes the importance of complications caused by metastatic disease to the remaining adrenal gland in patients with RCC who have undergone ipsilateral radical nephrectomy.

## Introduction

Renal cell carcinoma (RCC), the ninth most common neoplasm in the United States, accounts for 2% of global cancer diagnoses and deaths. Most cases of RCC are diagnosed incidentally on imaging, and mortality is dependent on the stage at diagnosis, with the metastatic disease having only a 12% five-year survival rate [1]. Metastatic spread can occur in almost any organ system, with the lungs, abdomen, bones, and brain the most commonly involved sites. Although juxtaposed to the kidney, the adrenal gland is uncommonly involved with metastasis to ipsilateral and contralateral adrenal, being around 3% and 0.7%, respectively [2]. We discuss the case of a 59-year-old male who presented to our tertiary care setup with atypical chest pain and altered mental status, who subsequently developed hypotension and hyponatremia, raising concern for adrenal insufficiency (AI). Further workup revealed the cause of adrenal hemorrhage due to contralateral metastasis, despite radical nephrectomy with ipsilateral adrenalectomy.

## Case presentation

**Figure 1:**
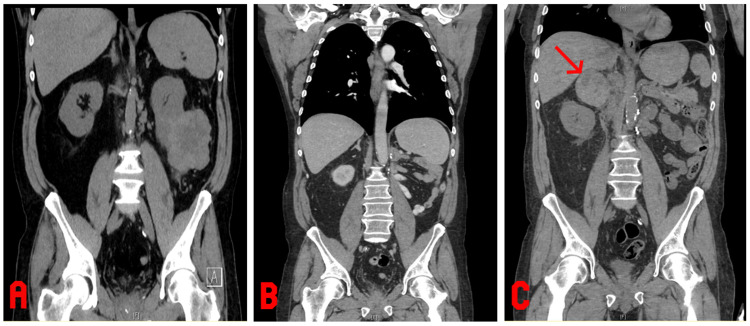
Computed tomography images. (A) Computed tomography of the abdomen/pelvis: noncontrast, coronal view, left-sided RCC; (B) Computed tomography of the chest/abdomen/pelvis: noncontrast, coronal view, post-left adrenalectomy and nephrectomy; (C) Computed tomography of the abdomen/pelvis: noncontrast, coronal view, new tumor invasion in the right adrenal gland with hemorrhage (red arrow). RCC: renal cell carcinoma

A 59-year-old male with a past medical history of chronic kidney disease, hypertension, inferior vena cava thrombus, pulmonary embolism on therapeutic-dose enoxaparin, and metastatic RCC, status post-left radical nephrectomy with ipsilateral adrenalectomy three years prior, presented to our tertiary care center with atypical chest pain. Computed tomography images showed left-sided RCC before the surgery (Figure 1A), after left adrenalectomy and left nephrectomy (Figure 1B), and new tumor invasion in the right adrenal gland with hemorrhage (Figure 1C).

Upon cancer diagnosis, the patient underwent left radical nephrectomy with ipsilateral adrenalectomy and was subsequently started on treatment with lenvatinib and everolimus, per standard protocol for metastatic RCC. The patient switched to sunitinib due to the development of acute renal failure. Follow-up positron emission tomography/computed tomography showed hypermetabolic foci in the liver and lungs, indicating disease progression despite treatment with sunitinib. The patient was taken off sunitinib and started on nivolumab but could not tolerate nivolumab due to drug toxicities. The patient then received cabozantinib one year prior to the presentation and was continued on it up to the current presentation.

The chest pain was new, atypical, sharp with radiation to the right side of the back and the right side of the abdomen, and rated a 9/10 on the pain scale. He denied any aggravating or alleviating factors, endorsed generalized weakness, and reported nonbloody, nonbilious emesis episodes. There were no other pertinent findings on the review of systems.

On arrival, vitals were as follows: blood pressure 182/105 mmHg, heart rate 104 beats per minute, respiratory rate 20 breaths per minute, oxygen saturation 98% on room air, and temperature of 36.3°C. The patient was in no acute distress; the physical examination was unremarkable, with clinical findings consistent with euvolemia. An electrocardiogram showed normal sinus rhythm. Initial labs were unremarkable (Table 1).

**Table 1:** Laboratory values during hospital admission. Hypotensive episode occurred on day three of the admission. COVID-19: coronavirus disease 2019; PCR: polymerase chain reaction

	Day 1	Day 2	Day 3	Day 4	Day 5	Day 6	Day 7	Day 8	Day 9	Day 10	References
White blood cell	6.9	5.2	5.8	8.0	8.5	6.0	5.1	5.4	6.0	6.8	3.50-10.80 K/uL
Hemoglobin	12.5	10.6	9.0	8.4	8.8	8.2	8.4	8.2	7.9	8.6	14-18 g/dL
Platelets	117	99	86	93	120	109	121	114	116	181	130-400 K/uL
Serum sodium	140	137	131	127	131	135	140	137	137	139	136-145 mmol/L
Serum potassium	4.7	4.0	4.2	4.5	4.0	4.0	3.8	3.6	3.7	3.7	3.5-5.1 mmol/L
Serum blood urea nitrogen	20	19	27	30	30	28	23	19	18	19	7-25 mg/dL
Serum creatinine	2.1	1.8	3.4	3.1	2.9	2.7	2.4	2.2	2.2	2.4	0.7-1.3 mg/dL
Troponin	0.02	-	-	-	-	-	-	-	-	-	<0.02 ng/mL
Heparin (Anti-Xa) level	1.59	-	-	-	-	-	-	-	-	-	1.0-2.0 U/mL
Aldosterone serum	-	-	<4.0	-	-	-	-	-	-	-	<21 ng/dL
Adrenocorticotropic hormone	-	-	524.9	-	-	-	-	-	-	-	7.4-64.3 pg/mL
Serum osmolality	-	-	271	-	-	-	-	-	-	-	280-295 mOsmol/kg
Morning cortisol	-	-	1.5	-	-	-	-	-	-	-	5.0-25.0 µg/dL
Thyroid-stimulating hormone	-	-	4.83	-	-	-	-	-	-	-	0.60-4.80 mIU/L
Blood culture	-	-	-	No growth to date	-	-	-	No growth to date	-	-	No growth to date
COVID-19 PCR RNA	Not detected	-	-	Not detected	-	-	Not detected	-	-	-	Not detected

Chest radiograph showed known metastatic lung disease. Ventilation-perfusion scan showed a known chronic right-sided pulmonary embolism, which had questionably increased in size, and a new perfusion defect in the lingula that may have signified a recent left-sided pulmonary embolism despite being on therapeutic-dose enoxaparin. The medical oncology service admitted the patient for further workup.

The hospital course became complicated by an abrupt drop in blood pressure and the onset of fever. Due to the concern for sepsis, the patient was pan-cultured, started on broad-spectrum antibiotics, and given a bolus of lactated ringer solution. A computed tomography scan of the abdomen and pelvis without contrast was obtained and revealed a new tumor invasion of the right adrenal gland with hemorrhage. The team stopped anticoagulation and repeated the bloodwork, which was significant for serum sodium of 127 mmol/L (normal range: 136-145 mmol/L), serum osmolality 249 mOsmol/kg (normal range: 285-295 mOsmol/kg), morning cortisol 1.5 µg/dL (5.0-25.0 µg/dL), and adrenocorticotropic hormone of 524.9 pg/mL (7.4-64.3 pg/mL), which were consistent with primary AI (Table 1).

The patient was given stress-dose steroids for hypotension and transitioned to daily fludrocortisone and hydrocortisone. His general condition improved remarkably with symptom resolution. After stabilization, we discharged the patient home with a hydrocortisone taper, which he tolerated well. On follow-up, he endorsed no complaints and remained hemodynamically stable.

## Discussion

We described a patient with known RCC who had undergone radical nephrectomy with ipsilateral adrenalectomy and subsequently presented with signs and symptoms of AI. Workup led to the diagnosis of AI secondary to adrenal metastasis to the contralateral adrenal gland in the setting of cabozantinib treatment. Typically, AI in RCC can be an autoimmune phenomenon, most commonly secondary to immunotherapy treatment. However, the clinical evidence of the case report demonstrates an alternative mechanism that, although rare, must be kept on the differential diagnosis.

In specific cohorts, AI can be a significant limiting factor in administering immune checkpoint inhibitors (ICPIs). ICPIs block the checkpoint molecules and promote immune tolerance, which is a double-edged sword. It can amplify T-cell response turning the immune system against tumor; simultaneously, unchecked immune tolerance can cause many inflammatory and autoimmune adverse effects, such as adrenalitis, presenting as AI [3-5]. For this reason, when a patient on an ICPI presents with symptoms concerning for AI, AI due to autoimmune etiologies is typically high on the differential diagnosis. However, this case presentation was an exception; the demonstration of metastatic disease on imaging raised the concern for metastatic etiology. Therefore, autoimmune etiology for AI was lower on the differential diagnosis, and hence further workup of adrenalitis was determined to be not indicated at that time.

RCC is notorious for causing late metastasis. Contralateral adrenal metastasis has been reported in the literature as late as 23 years post-RCC diagnosis; among various RCC subtypes, most reports show clear cell histology [6]. Late contralateral adrenal metastasis recurrence following radical nephrectomy has only been documented in a handful of cases and is theorized to be due to one of the two primary reasons [7]: the slow growth of tumors in the case of low-grade tumors and loss to follow-up with regular surveillance imaging.

Different proposed mechanisms explain adrenal metastasis, such as antegrade hematogenous spread versus antegrade venous spread, although the literature is sparse in characterizing the two mechanisms. In addition, most metastases to adrenal glands are asymptomatic, but a handful of cases have identified AI. Thus, AI features should alert physicians of underlying adrenal metastasis in patients with prior RCC irrespective of the timeline of nephrectomy, as was the case in our patient, emphasizing regular surveillance imaging in patients with RCC of disease remission duration [8].

Magnetic resonance imaging or computed tomography with and without contrast is the preferred modality for detecting tumor burden in the abdomen along with continued surveillance screening, with frequency and duration of surveillance depending on tumor stage, risk factors, and patient characteristics [9]. Despite the previous urological dogma for RCC management, there have been increasing questions regarding the need for adrenalectomy. Concerning the ipsilateral adrenal gland in RCC, multivariate analysis has established that tumor size is predictive of adrenal involvement rather than tumor presence at the upper pole, with recommendations to avoid adrenalectomy in radical or partial nephrectomy cases, thereby avoiding the risk of Addisonian crisis [9].

The Metastatic Renal Cancer Database Consortium risk model is used to determine the treatment modality for metastatic RCC [10]. Through this approach, there is stratification for systemic chemotherapy. However, recent trials have made a paradigm shift in treatment-naïve RCC, with ICPI combination targeted therapies now becoming the mainstay of treatment. For instance, the KEYNOTE-426 [11] and Checkmate-214 [12] trials showed the superiority of axitinib in combination with pembrolizumab and nivolumab and ipilimumab over previously established therapy with tyrosine kinase inhibitor sunitinib. Similarly, the CABOSUN phase II randomized trial demonstrated equal efficacy of cabozantinib compared to sunitinib in the treatment of RCC in patients with intermediate and poor risk [3,4].

AI is a medical emergency that can present as refractory hypotension requiring steroid replacement and electrolyte monitoring. Therefore, it should be included in the differential diagnosis in any patient receiving treatment for or with a history of RCC [5]. However, it is essential to recognize AI in these patients, diagnose the etiology correctly, and treat based on the etiology, as treatment differs based on the etiology. Adrenalitis, an autoimmune reaction, can be treated with stress-dose steroids. AI due to metastases or hemorrhage, however, requires treatment of the malignancy. Furthermore, physicians must evaluate for metastases and disease recurrence in patients with a history of RCC, regardless of remission status, who present with AI.

## Conclusions

Primary AI due to metachronous metastatic disease of the contralateral adrenal from RCC is very rare. As described here, although rare, RCC can recur or metastasize to the remaining contralateral adrenal gland after nephrectomy with adrenalectomy causing primary AI. This case emphasizes the importance of a thorough history, focused physical examination, broad differential diagnosis, and complete workup for RCC patients who present with nonspecific symptoms.
